# Supplementary material for: Association between Influenza Vaccination and the Risk of Bell’s Palsy in the Korean Elderly
Source: Vaccines (Basel). 2021 Jul 6;9(7):746. doi: 10.3390/vaccines9070746 (PMC8310133; doi:10.3390/vaccines9070746)
Supplement: Supplementary file 1 [file vaccines-09-00746-s001.zip › Supplementary.pdf]

vaccines

MDPI

Subgroup	Risk interval: Days 1-14, Control interval: Days 29-42		Risk interval: Days 1-28, Control interval: Days 43-70	
	No. of cases in risk/control interval	Incidence Rate Ratio (95% CI)	No. of cases in risk/control interval	Incidence Rate Ratio (95% CI)
**Total**	475/441	1.08 (0.95-1.23)	907/866	1.05 (0.95-1.15)
**Age**				
65-74	286/268	1.07 (0.90-1.26)	569/522	1.09 (0.97-1.23)
75-84	162/153	1.06 (0.85-1.32)	299/301	0.99 (0.85-1.17)
Over 85	27/20	1.35 (0.76-2.41)	39/43	0.91 (0.59-1.40)
**Gender**				
Female	281/248	1.13 (0.96-1.34)	518/497	1.04 (0.92-1.18)
Male	194/193	1.01 (0.82-1.23)	389/369	1.05 (0.91-1.22)
**Time of vaccination**				
September	64/67	0.96 (0.68-1.35)	112/108	1.04 (0.80-1.35)
October	380/354	1.07 (0.93-1.24)	742/709	1.05 (0.94-1.16)
November	26/18	1.44 (0.79-2.63)	46/41	1.12 (0.74-1.71)
**Comorbidities**				
Diabetes	145/108	1.34 (1.05-1.72)	243/236	1.03 (0.86-1.23)
Dyslipidemia	102/75	1.36 (1.01-1.83)	180/151	1.19 (0.96-1.48)
Hypertension	239/219	1.09 (0.91-1.31)	453/425	1.07 (0.93-1.22)

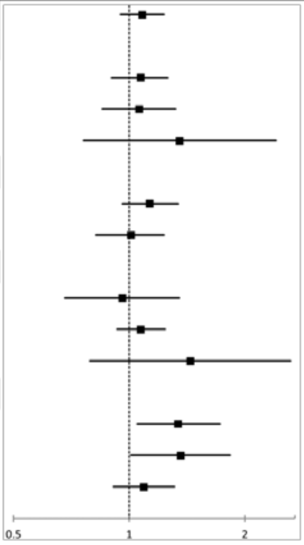

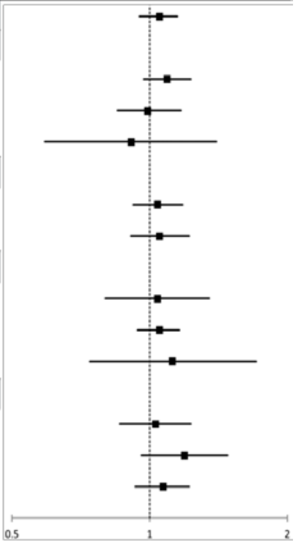

Figure S1. Subgroup analysis for the risk of Bell's palsy after influenza vaccination in sensitivity analyses.

vaccines

MDPI

Table S1. Baseline characteristics of Bell's palsy cases after influenza vaccination for sensitivity analyses.

Characteristics	Cases in risk interval Days 1-14 post-vaccination		Cases in control interval Days 29-42 post-vaccination		*p*	Cases in risk interval Days 1-28 post-vaccination		Cases in control interval Days 43-70 post-vaccination		*p*
	N	(%)	N	(%)		N	(%)	N	(%)	
**Total**	475	(100%)	441	(100%)		907	(100%)	866	(100%)	
**Age at vaccination**										
65-74	286	(60.21%)	268	(60.77%)	0.7322	569	(62.73%)	522	(60.28%)	0.5275
75-84	162	(34.11%)	153	(34.69%)		299	(32.97%)	301	(34.76%)	
Over 85	27	(5.68%)	20	(4.54%)		39	(4.30%)	43	(4.97%)	
**Gender**										
Female	281	(59.16%)	248	(56.24%)	0.3710	518	(57.11%)	497	(57.39%)	0.9055
Male	194	(40.84%)		(43.76%)		389	(42.89%)	369	(42.61%)	
**Months of vaccination**										
Jan-Feb	1	(0.21%)	0	(0%)		1	(0.11%)	4	(0.46%)	
Mar-Apr	0	(0%)	0	(0%)	0.2767[a]	0	(0%)	0	(0%)	0.3699[a]
Sep-Oct	444	(93.47%)	421	(95.46%)		854	(94.16%)	817	(94.34%)	
Nov-Dec	30	(6.32%)	12	(4.33%)		52	(5.73%)	45	(5.20%)	
**Type of vaccinated institution**										
Private health institution	400	(84.21%)	375	(85.03%)	0.7300	761	(83.90%)	714	(82.45%)	0.4128
Public health institution	75	(15.79%)	66	(14.67%)		146	(16.10%)	152	(17.55%)	
**Vaccinated regions**										
Capital region	188	(39.58%)	160	(36.28%)	0.3349	358	(39.47%)	363	(41.92%)	0.3915
Metropolitan city	126	(26.53%)	111	(25.17%)		233	(25.69%)	200	(23.09%)	
Others	161	(33.89%)	170	(38.55%)		316	(34.84%)	303	(34.99%)	
**Setting of diagnosis**										
Inpatient	103	(21.68%)	107	(24.26%)	0.3535	188	(20.73%)	183	(21.13%)	0.8344
Outpatient	372	(78.32%)	334	(75.74%)		719	(79.27%)	683	(78.87%)	

Table S2. Risk of Bell's palsy after influenza vaccination by the month of vaccination

Time period [*]	No. of cases in risk interval[†]	No. of cases in control interval[‡]	IRR (95% CI)	No. of cases in risk interval[§]	No. of cases in control interval[‖]	IRR (95% CI)	No. of cases in risk interval[¶]	No. of cases in control interval[#]	IRR (95% CI)
**Total**									
September	64	67	0.96 (0.68-1.35)	112	108	1.04 (0.80-1.35)	179	181	0.99 (0.80-1.22)
October	380	354	1.07 (0.93-1.24)	742	709	1.05 (0.94-1.16)	1.096	1.102	0.99 (0.91-1.08)
November	26	18	1.44 (0.79-2.63)	46	41	1.12 (0.74-1.71)	14	68	0.94 (0.67-1.32)
December	4	2	2.00 (0.37-10.92)	6	4	1.50 (0.42-5.32)	8	6	1.33 (0.46-3.84)
**2015/2016 season**									
September	1	0	-	1	1	1.00 (0.06-15.99)	1	1	1.00 (0.06-15.99)
October	131	125	1.05 (0.82-1.34)	245	284	0.86 (0.73-1.02)	370	399	0.93 (0.80-1.07)
November	4	4	1.00 (0.25-4.00)	9	14	0.64 (0.28-1.49)	13	19	0.68 (0.34-1.39)
December	2	0	-	3	2	1.50 (0.25-8.98)	3	2	1.50 (0.25-8.98)
**2016/2017 season**									
September	0	1	-	0	0	-	1	0	
October	147	147	1.00 (0.80-1.26)	301	263	1.14 (0.97-1.35)	448	422	1.06 (0.93-1.21)
November	5	5	1.00 (0.29-3.45)	10	12	0.83 (0.36-1.93)	15	18	0.83 (0.42-1.65)
December	1	2	0.50 (0.05-5.51)	1	1	1.00 (0.06-15.99)	3	2	1.50 (0.25-8.98)
**2017/2018 season**									
September	63	66	0.95 (0.68-1.35)	111	107	1.04 (0.80-1.35)	177	180	0.98 (0.80-1.21)
October	102	82	1.24 (0.93-1.66)	196	162	1.21 (0.98-1.49)	278	281	0.99 (0.84-1.17)
November	17	9	1.89 (0.84-4.24)	27	15	1.80 (0.96-3.38)	36	31	1.16 (0.72-1.88)
December	1	0	-	2	1	2.00 (0.18-22.06)	2	2	1.00 (0.14-7.10)

Abbreviations: IRR, incidence rate ratio; CI, confidence interval.

[*] Overall, the doses of vaccinations between January and April were rare, and the number of patients who were vaccinated during the period and included in the risk and control

vaccines

MDPI

intervals was very small, so they were omitted from the table. †Days 1-14 post-vaccination. ‡Days 29-42 post-vaccination. §Days 1-28 post-vaccination. ‖Days 43-70 post-vaccination. ¶Days 1-42 post-vaccination. #Days 57-98 post-vaccination.
